# A Study of Coronary Stent Thrombosis Growth

**DOI:** 10.3390/bioengineering13070779

**Published:** 2026-07-07

**Authors:** Ivan Fernney Ibanez Aguilar, Marcos Beltrão Paraizo, Pedro Soares Teixeira, Adriano Camargo Carneiro, Bruno Alvares de Azevedo, Angela Ourivio Nieckele, Carlos Eduardo Rochitte

**Affiliations:** 1 Hospital do Coração—HCor, São Paulo 04004-030, SP, Brazilrochitte@cardiol.br (C.E.R.); 2 Department of Mechanical Engineering, Pontifical Catholic University of Rio de Janeiro, Rio de Janeiro 22451-900, RJ, Brazilnieckele@puc-rio.br (A.O.N.); 3 Fit Center—Clínica de Performance Humana, Niterói 24220-331, RJ, Brazil

**Keywords:** coronary, stent thrombosis, computational fluid dynamics

## Abstract

In this study, the impact of thrombus growth at the left coronary ostium—resulting from a misplaced stent—was investigated through numerical simulations. Blood flow from the aortic root into the coronary arteries was modeled for several potential thrombus sizes to assess changes in pressure and shear stress caused by thrombus development. The results showed that even in the most severe case examined (with the thrombus growing three times its original size), the obstruction to the coronary cross-sectional area was not sufficient to produce a significant pressure drop or wall shear stress increases. This outcome is primarily attributed to the thrombus’s location at the tip of the stent; even with considerable elongation, it did not significantly narrow the coronary artery’s lumen. While the specific configurations studied did not result in critical obstruction, it is important to note that other thrombus shapes or positions could potentially block flow more severely, leading to substantial pressure drops in the left coronary arteries.

## 1. Introduction

Stent thrombosis (ST) incidents have been considerably reduced after the introduction of the second-generation “Drug Eluting Stents”, as shown in clinical trials. When ST occurs, it is often associated with catastrophic outcomes, such as acute myocardial infarction or sudden cardiac death, underscoring the importance of continued surveillance and prevention strategies in stented patients [1,2]. Advances in stent technology and the advent of second-generation “Drug Eluting Stents” have reduced the incidence of ST to <1% per year, but have not eliminated it [3]. A misplaced stent can induce thrombus formation among other problems and surgical removal is recommended [4]. Depending on the physical and chemical characteristics of the stent, the ST occurrence rate can vary substantially. Tada et al. [5] presented the ST occurrence rate in terms of different types of stents after 3 years of being installed: 2.2% in the case of early-generation drug-eluting stents, 1.5% when using bare-metal stents, and 1.0% for new-generation drug-eluting stents. Another possibility to reduce the ST occurrence rate is by antithrombotic drugs (anticoagulants and antiplatelet drugs) [6]. In terms of the percentages mentioned above, ST can be considered as a rare event, although, since ST appears, the mortality rate can reach 40% [7]. With such high mortality rates, ST is a clinical complication that has been studied since the first implantation of a coronary stent in 1986 [8]. Stent thrombosis can be identified through computed tomography angiography or, unfortunately, after acquiring post-mortem evidence [9]. Reports have shown that specific patient characteristics (e.g., presentation with an acute coronary syndrome, history of diabetes, impaired left ventricular systolic function, impaired platelet reactivity) and lesions with high anatomical complexity are associated with a higher incidence of ST.

The development of an aortic root thrombosis (ART) after a left ventricular assist device (LVAD) procedure has been reported by Fried et al. [10], who found that ART is associated with significant morbidity and mortality. They emphasized the need for further research into the clinical implications of ART in LVAD patients. Similarly, Veenis et al. [11] evaluated a small number of patients with ART after a Transcatheter Aortic Valve Implantation (TAVI) or LVAD. Their findings suggest that early anti-thrombotic therapy and vigilant diagnostic follow-up can be a satisfactory strategy to prevent the emergence of ART. They also mentioned the need for more studies regarding the consequent risk of ART after TAVI and LVAD. Stent thrombosis can also occur one to five years after stent implantation [12]. Although rare, it remains a serious and concerning complication. To prevent acute and sub-acute ST after longitudinal stent deformation caused by a stuck catheter, it has been proposed to implant a bailout stent [13]. After conducting a bibliographic search, no numerical or experimental studies regarding aortic root thrombosis (ART) resulting from a coronary stent implantation were found. Nevertheless, some studies related to the broader context of the present work have been reported in specialized literature. For instance, the relationship between thrombus formation and inadequate stent positioning within coronary arteries has been investigated through numerical approaches in recent years, as illustrated by the studies of Qu et al. [14] and Wei et al. [15]. In the work conducted by Qu et al. [14], thrombus formation resulting from different stent malposition configurations inside the coronary artery was modeled by solving the advection–diffusion equation governing the transport mechanisms associated with thrombus generation. On the other hand, the study by Wei et al. [15] focused on the analysis of hemodynamic patterns associated with different stent malposition conditions within the coronary artery. Their work primarily investigated wall shear stress-related hemodynamic metrics on the stent and arterial walls, without explicitly modeling thrombus formation. This absence highlights a clear gap in current knowledge that warrants further investigation. Stent thrombosis within the aortic root still lacks reference values that indicate the presence or absence of ischemia. Therefore, both clinical management with medication (anticoagulants and antiplatelet agents) and interventional management (repeat angioplasty or open-heart surgery) still lack data for assertive decision-making. Thus, this article presents a first approach to quantifying blood flow in these conditions and inferring hemodynamic consequences with the progression of the thrombotic lesion. Considering the potential risk of the ST on the patient’s quality of life, this case report numerically investigated the impact of a possible growth of the thrombus.

## 2. Materials and Methods

This study is a case report involving a 65-year-old male with previously known coronary disease and a stent implanted a few years earlier in the left descending coronary artery. He underwent computational tomography angiography (Angio-CT) in March 2016 as an investigation for coronary disease. The exam showed an obstruction on the ostium of the main left coronary, suggestive of a 70% lesion. In April 2016, a cardiac catheter was inserted with angiography, and intravascular ultrasonography was performed. This exam revealed a 50% obstruction of the ostium of the main left coronary artery. Thus, a stent Biomatrix 4.0/11 mm was implanted, with adequate expansion and post-dilatation with a 4.5/8 mm balloon, presenting a satisfactory result at the after-procedure angiography.

**Figure 1:**
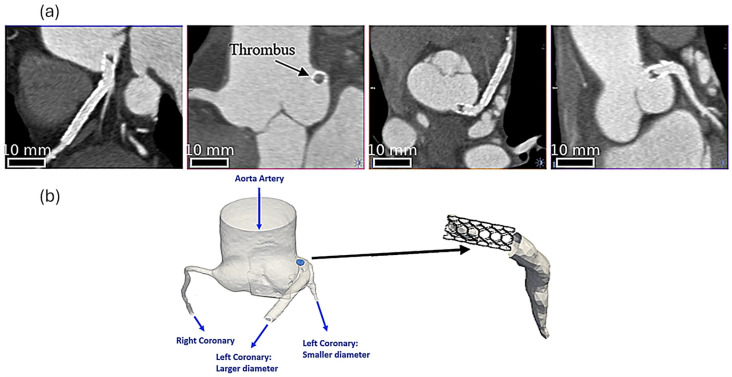
Coronary  artery and stent representation: (**a**) Angio-CT; (**b**) aortic root.

Three years later, with no symptoms related to coronary disease, and as part of the institution’s clinical routine, the patient underwent a pharmacological stress test with scintigraphy and another angio-CT of the coronary arteries. The scintigraphy results revealed a decreased and persistent uptake of radioisotope in the inferior lateral wall of the left ventricle with no signs of ischemia related to the administration of dipyridamole or a normal ejection fraction. Angio-CT (Figure 1a) revealed a main angulated left coronary artery and a stent in the ostium with its proximal border localized in the aortic root, as well as a hypodense image leading to luminal reduction, and this misplaced stent led to the emergence of a thrombus. Nevertheless, contrary to what was expected [16], the patient did not present any symptoms. Thrombus removal by aspiration has been employed, but it is challenging and can lead to surgical emergency and the need for coronary bypass [17]. This confirms the importance of monitoring thrombus formation after a good stent expansion, as reported by Murakami et al. [18]. The thrombus likely developed at the reported location as a consequence of the hemodynamic patterns arising from the interaction between the stent and the blood flow. The portion of the stent positioned at the aortic root acts as a barrier, altering blood flow patterns during coronary filling in the diastolic phase. Prolonged interaction between the stent-induced barrier and regions of low velocity and flow recirculation may have increased local blood viscosity. This effect was more pronounced in the segment farther from the entrance of the left coronary artery, contributing to thrombus formation.

The patient was already taking oral anticoagulants due to permanent atrial fibrillation; therefore, no new therapy was added to this case. At the time of publication of this article, the patient remains asymptomatic and has been advised to return annually to assess the need for a new coronary artery CT angiography.

Figure 1b illustrates the computational aortic root and coronary artery, as well as the stent representation. No influence of the ST on the patient’s health was identified, possibly because it was not located within the left coronary artery. Nevertheless, the presence of an ST constitutes a potential clinical concern, as it may lead to severe complications. Given that the hemodynamic impact of an ST located outside the coronary artery remains unknown, the present study seeks to numerically assess the influence of the appearance and possible growth of this ST on hemodynamic variables, especially in the pressure field in the vicinity of the coronary arteries and aortic root.

**Figure 2:**
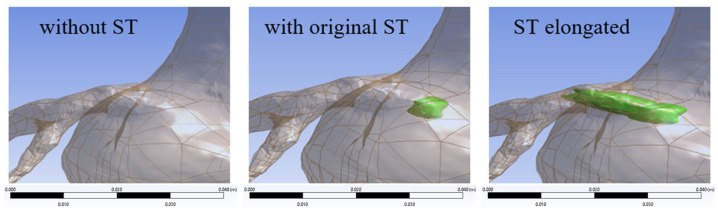
Domain geometry: without ST; ST with original size (∅2.3 mm × 4.2 mm); with elongated ST (∅2.3 mm × 13.0 mm).

Here, three different scenarios of coronary stent thrombosis that occurred within the aortic root (Figure 2) were evaluated by analyzing the pressure and WSS (wall shear stress) distribution, which were numerically determined by the solution of the flow field conservation equations. The ST regions correspond to the green structures highlighted in Figure 2. The objective of these simulations was to evaluate the hemodynamic impairment of thrombosis in its current state and how it may evolve if the thrombus grows. In this study, the presence of the stent geometry was not considered since the local hemodynamic effects associated with the stent geometry would likely become more relevant in simulations focused on thrombus initiation and evolution mechanisms, particularly in studies involving species transport, blood phase transition phenomena, platelet activation, or thrombus growth dynamics.

### 2.1. Geometry: Segmentation and the 3D Model

The first step of the present analysis was to build a numerical domain from the patient images of an angio-CT scan. Figure 1b illustrates the resulting domain created, where the thrombus can be seen at the entrance of the left coronaries.

The aortic root and coronary arteries geometries were built based on an angio-CT scan obtained with a 64-slice scanner SOMATOM Sensation 64 (Siemens, Germany). The selected angio-CT slices spanned from the aortic annulus to the beginning of the ascending aorta (see Figure 1a). The angio-CT data used for segmentation presented a spatial resolution of 0.60 mm/slice along the Z direction and approximately 0.26 mm/pixel in the XY plane. The image segmentation was performed with the software FIJI (Fiji Is Just ImageJ, version 1.53c), an open-source image processing software, focusing on biological image analysis [19]. Figure 1b illustrates the resulting domain created, where the thrombus can be seen at the entrance of the left coronaries. In the present simulations, the thrombus was modeled as a rigid impermeable obstruction incorporated into the computational geometry, and no flow was allowed through the thrombus region.

Details of the domain geometry at the thrombus region of the three configurations examined (without thrombus, with the original-sized thrombus observed in the CT angiography, and the elongated thrombus) are illustrated in Figure 2. Note that the stent was not modeled.

### 2.2. Flow Modeling

In the sequence, to determine the flow field within the aortic root portion and coronary arteries in the presence of the patient’s pathology, the following hypotheses were considered to define the mass and momentum conservation equations.

Considering that the coronary arteries are irrigated only in the diastolic period, the aortic valve was assumed to be closed. To simplify the problem and aim to investigate the most extreme situation for the patient, a steady flow was imposed entering the aorta (Figure 1b). The effects of gravity were neglected since the pressure variations are dominant over the force of gravity. The blood was considered an incompressible fluid [20], and the density was set at ρ = 1054 kg/m^3^ [21]. The blood in the aortic root and coronary arteries was modeled as a non-Newtonian fluid [22]. Although there is still ongoing discussion in the literature regarding the necessity of modeling blood as a non-Newtonian fluid in arteries with diameters larger than 0.5 mm [23,24], non-Newtonian models may still improve the local representation of blood flow even in larger arteries (~3 mm), particularly in regions characterized by recirculation, low shear rates, and areas near thrombus formation. Nevertheless, the impact of modeling the flow as Newtonian and non-Newtonian was examined by Ibanez et al. [25], and a very small difference between the results was obtained. In light of these considerations, a conservative approach was adopted in the present study by accounting for the non-Newtonian behavior of blood flow. The Carreau model [26] was selected as the fluid constitutive equation, as recommended by Narayan et al. [27] and Wajihah and Sankar [28]. The blood flow descending through the ascending aorta (Figure 1b) was considered laminar, corresponding to a Reynolds number equal to 55. The resulting conservation of mass and momentum equations are

(1) $$\frac{\partial u_{j}}{\partial \;x_{j}}=0\;;\;\;\;\;\;\;\frac{\partial \rho \;u_{j}}{\partial \;x_{j}}+\frac{\partial {\;\rho \;u_{i}\;u}_{j}}{\partial \;x_{j}}=-\frac{\partial p}{\partial \;x_{i}}+\frac{\partial \;\left(\tau_{ij}\right)}{\partial \;x_{j}}$$

where $u_{j}$ and $x_{j}$ are the velocity components and coordinate system, respectively. Note that the repeated subscript implies Einstein summation ($a_{i}b_{i}=\sum_{i=1}^{3}a_{i}b_{i}$). p is pressure, and $\tau_{ij}$ is the viscous stress, which is proportional to the deformation rate tensor $S_{ij}$, and the viscosity η is defined as

(2) $$\tau_{ij}=\eta \;2\;S_{ij}\;;\;\;2\;S_{ij}=\left(\frac{\partial u_{i}}{\partial \;x_{j}}+\frac{\partial u_{j}}{\partial \;x_{i}}\right);\;\;\;\;\eta =\mu_{\infty }+\left(\mu_{0}-\mu_{\infty }\right){\left[1+{\left(\lambda \;\dot{\gamma }\right)}^{2}\;\right]}^{\frac{n-1}{2}}$$

In Equation (2), $\dot{\gamma }=\sqrt{2S_{ij}S_{ij}}$ is the deformation rate modulus (or simply shear rate), $\mu_{\infty }$ = 3.45 cP is the viscosity at high strain rates, and $\mu_{0}=$ 56 cP is the lower limit corresponding to low strain rates. λ = 3.313 s and n=0.3568 are adjustable parameters of the Carreau model.

### 2.3. Boundary Conditions

**Figure 3:**
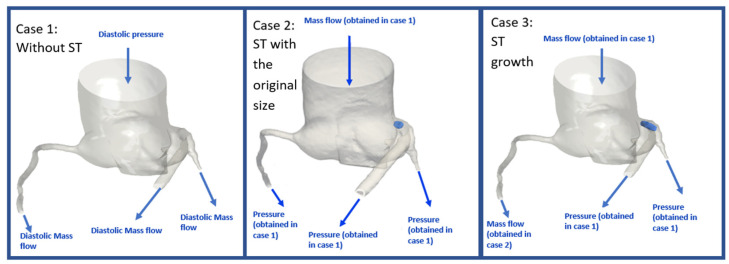
Schematic representation of the boundary conditions for each case.

The strategy adopted in this study to identify the hemodynamic pattern evolution due to the thrombus presence was based on three different situations (Figure 2): (i) before the ST appearance; (ii) ST with the original size identified in the angio-CT (4.2 mm); and (iii) elongated ST (artificially increased ST size in the longitudinal direction of the stent). For each of the evaluated scenarios, the reasoning of the boundary conditions used is shown schematically in Table 1 and Figure 3. The ST regions correspond to the blue structures highlighted in the Figure 3. Regarding Case 1 (without the presence of ST), physiological pressure and mass flow rate for a healthy patient are well defined in the literature [29,30]. This first case was investigated to determine the pressure at the left and right coronaries for a healthy patient.

The output pressures in left and right coronaries obtained in Case 1 were imposed as boundary conditions for Case 2. The reason for this is that after ST formation, the patient remained asymptomatic. Input mass flow rate obtained in Case 1 was used as boundary conditions for Case 2 because the mass flow rate at the aortic root input will not be affected by ST formation since it is located substantially far from the ST.

Between Cases 2 and 3, only the boundary condition in right coronary was changed. Since the ST growth impact is necessarily observed in the left coronary artery, near the region where the ST was formed, it was assumed that the flow rate (and head loss) along the right coronary was not changed. The strategy used to assess the ST influence was based on the consideration that the ST growth impacts only the flow rate (and head loss) in the left coronaries (i.e., localized pressure difference between the region of the aortic root immediately before the ST and the region of the left coronary immediately after the ST).

### 2.4. Numerical Modeling

The present analyses were performed using ANSYS Fluent v20 R1 software [31], which solves the conservation equations discretized based on the finite volume method [32]. The discretized equations were obtained with the “2nd order Upwind” discretization scheme for the spatial terms. To handle non-linearities, a Picard iterative method was applied, with under-relaxation factors equal to 0.3 and 0.7 for pressure and momentum equations, respectively. For the solution of the pressure and velocity coupling, the coupled algorithm available in Fluent was used. The discretized equations were solved by the line-by-line Gauss–Seidel algorithm combined with the additive multigrid method. To guarantee convergence, the tolerance of the residual error was set as $10^{-12}$ for all equations.

**Figure 4:**
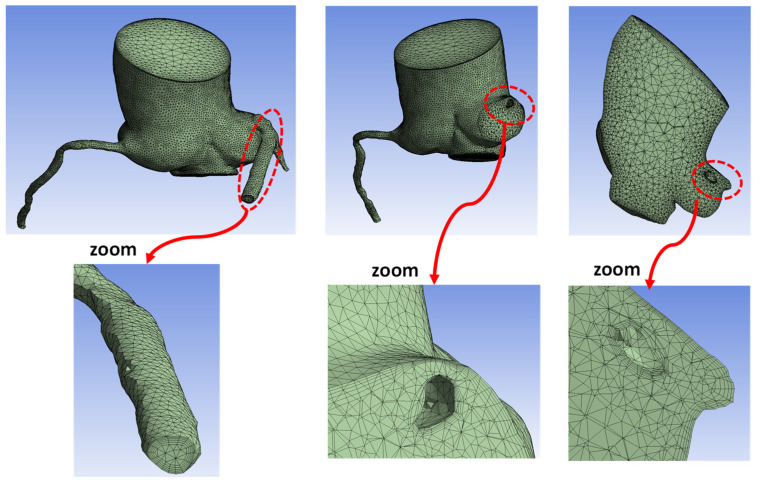
Details of the computational mesh of the aortic root and coronary arteries. Case: original ST size.

A mesh with 260,000 nodes was applied to all the cases studied; as an example of the computational mesh quality, Figure 4 illustrates the mesh employed for the case corresponding to the original ST size. The computational mesh was generated using an unstructured tetrahedral element approach. Larger elements were used in regions where smoother gradients of the resolved variables were expected, while special attention was dedicated to the refinement of regions near the walls of the aortic root, coronary arteries, and especially in the vicinity of the thrombus surface, where stronger gradients of pressure, velocity, and wall shear stress were anticipated.

To improve the mesh resolution in these critical regions, the inflation layer method available in the ANSYS Meshing software was employed. Specifically, five inflation layers with a growth rate of 1.2 were applied near the walls. Besides providing the necessary local refinement, the inflation layers generate elements aligned normally to the wall boundaries, which improves the numerical treatment of near-wall gradients and facilitates the stability and accuracy of the simulations. The resulting mesh presented an average spatial resolution of approximately 1.5 mm^3^ per element.

The grid distribution was determined based on a mesh independence test, employing the Grid Convergence Index, $GCI=F_{s}\varepsilon_{\phi }/\left(r^{p}-1\right)$ [33], where $F_{s}=1.25$ is the safety factor as recommended by Elsayed and Lacor [34], $r=\Delta x_{n}/\Delta x_{n+1}$ is the mesh ratio, p=2 corresponds to a second-order method, and $\varepsilon_{\phi }=\left|\left(\phi_{n}-\phi_{n+1}\right)/\phi_{n+1}\right|$ is the relative error of ϕ (variable considered). Table 2 shows that GCI for the pressure drop $\left(\Delta P_{lc}\right)$ and pressure at the inlet $\left(P_{in,lc}\right)$ of the left coronary (lc) were smaller than 1.6% for the mesh selected.

Simulations were post-processed with ANSYS CFD-Post tool and with the open-source Paraview.

## 3. Results

**Figure 5:**
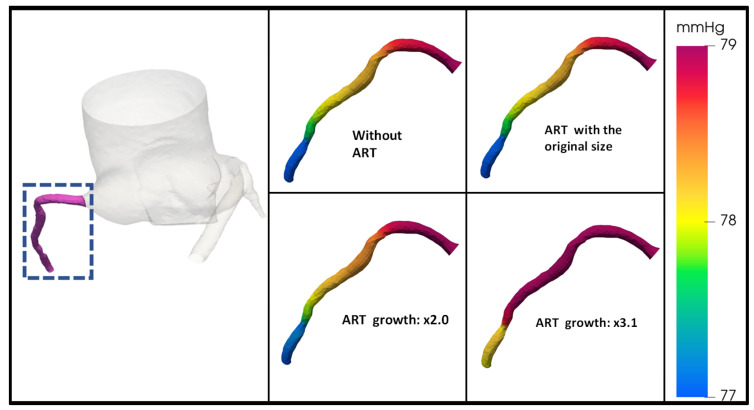
Pressure distribution along the right coronary.

First, the impact of the ST on the pressure distribution on the right coronary wall is shown in Figure 5. During the thrombus growth, the same mass flow rate was imposed for all cases at the right coronary, and as a result, the pressure drop along the right coronary was also the same, approximately 2 mmHg. Note, however, that the presence of a thrombus leads to an increase in the pressure level. For the case where the ST length grew by a factor of three (x3.1), the pressure level increased by 1 mmHg.

**Figure 6:**
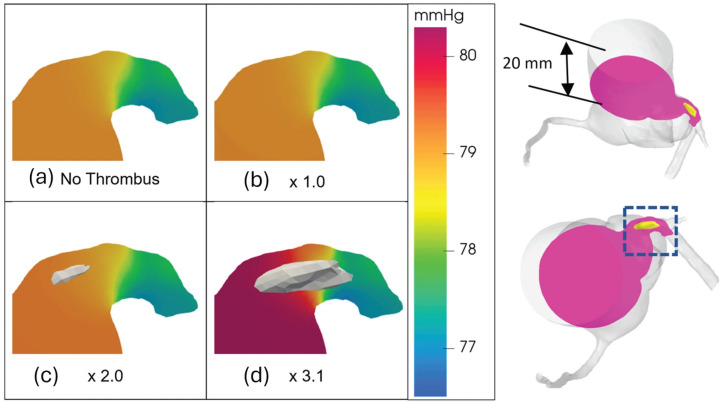
Pressure field in the ST vicinity. (**a**) No ST; (**b**) original ST = 4.2 mm; (**c**) increased ST by 2 = 8.4 mm; (**d**) increased ST by 3.1 = 13 mm.

The obstruction caused by the ST growth in the entry region of the left coronary artery is examined in Figure 6, which illustrates the pressure field in a parallel plane to the aorta artery input, with a displacement equal to 20 mm. Note that the ST is only visible in the selected plane when the ST has increased by ×2.0. Between the upstream and downstream regions of the ST, it is possible to observe an increasing pressure drop due to the ST growth (of approximately 3 mmHg in the most extreme case).

**Figure 7:**
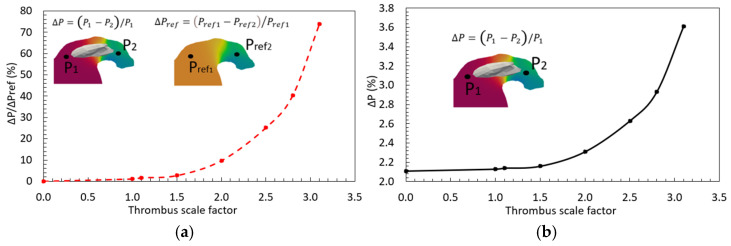
(**a**) Increase in relative pressure drop ΔP/ΔP_ref_(%). (**b**) Pressure drop ΔP evolution in each case.

Figure 7a shows the pressure drop (ΔP) due to the thrombus, corresponding to each case in relation to the pressure drop without ST ($\Delta P_{ref}$), which was equal to 220 Pa (1.65 mmHg). A relative pressure drop ($\Delta P/\Delta P_{ref}$) increase of 73.9%, corresponding to the maximum ST growth, was obtained. However, the pressure drop from upstream to downstream of the ST in relation to the pressure level upstream of the ST is less expressive, as shown in Figure 7b, varying between 2% and 3.6%, as the ST is elongated.

**Figure 8:**
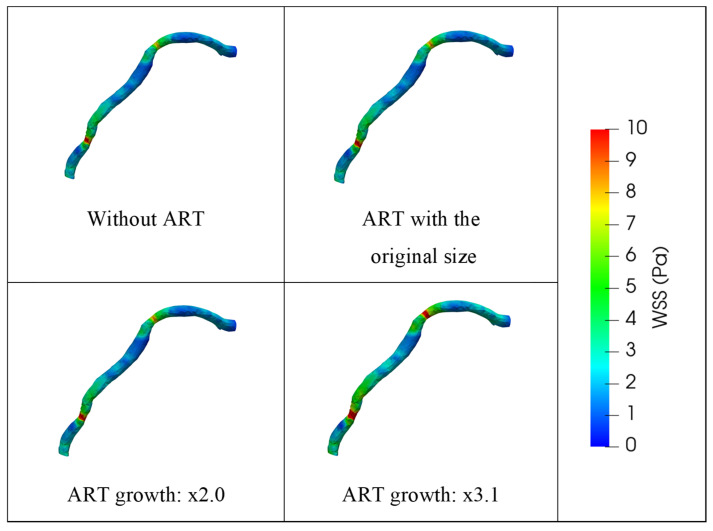
WSS distribution along the right coronary.

Regarding the effects of thrombus growth on the wall shear stress (WSS) in the coronary arteries, Figure 8 shows the WSS field in the right coronary artery for the four simulated cases: without ST, with an ST of normal size, and with an enlarged ST of 2× and 3.1× factors. The figure indicates that the appearance and growth of the ST up to twice its original size does not significantly affect the levels or distribution of WSS in the right coronary artery. However, when the thrombus size is increased to 3.1 times its original volume, a slight expansion of regions exposed to higher WSS values can be observed, particularly in areas where the vessel diameter narrows and a sharp curvature begins.

**Figure 9:**
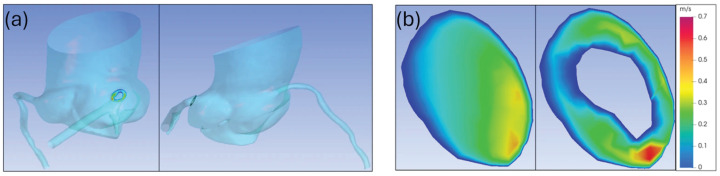
(**a**) Plane used to measure area obstruction at the left coronary input. (**b**) Effect of ST obstruction at the left coronary input.

Figure 9a presents two different views of the location of the plane employed to estimate the flow-obstructed area. Figure 9b illustrates the cross-sectional area of the referred plane without and with ST. Without ST, the measured area is 25.2 mm^2^, while the cross-sectional area of the same plane in the presence of the longest ST (×3.1) is 18.4 mm^2^, that is, around 27% smaller than its original size.

## 4. Discussion

The main objective of the present study was to identify the possible impact of the ST growth on the pressure in the coronary arteries. For the case where the ST length increased by a factor of three (×3.1), an increase in the pressure level in the right coronary artery was noted, suggesting that this was to maintain the same amount of mass flow rate flowing through it, reflected by an increase of 1 mmHg in the pressure distribution. An increase in the pressure gradient with ST in relation to without ST ($\Delta P/\Delta P_{ref}$), corresponding to the maximum ST growth, demonstrates that as the ST is elongated, it can cause noticeable hemodynamic alterations.

A pressure drop from upstream to downstream of the ST in relation to the pressure level upstream of the ST (smaller than 3.6%) was noticed. Vianna [35] has shown that for a 50% coronary obstruction, the pressure drop ΔP can be approximately 25%, which can already be considered as a type of ischemia [36]. Currently, we do not have a reference threshold to define ischemia in an ST inside the aortic root. Therefore, we use the literature value [37] for intracoronary lesions, in which a pressure drop greater than 20% on FFR CT defines the presence of an ischemic lesion. The maximum ST growth (×3.1) towards the left coronary did not generate a coronary obstruction high enough to produce pressure drops related to the onset of ischemia. The previous statement should be evaluated with caution since this study did not consider possible random ST growths.

The obtained pressure drop along the ST (smaller than 4% for the longest ART of ×3.1) was in accordance with the observation of Vianna [35] since the area reduction corresponding to the most extreme case of the elongated ST evaluated in this study was approximately half of the obstruction size associated with the significant pressure drop reported by Vianna [35].

## 5. Study Limitations

One of the limitations of the present study is the exclusion of the systolic phase of coronary blood flow and the associated variations in myocardial resistance during ventricular contraction. The steady-state analysis neglects physiological effects due to the transit regime, which may lead to a systematic underestimation of the hemodynamic impact of the intravascular thrombus. Consequently, the results should be interpreted with caution, as the adopted assumptions may not fully capture the complex transient interactions between coronary flow dynamics and myocardial contraction.

Another limitation is the simplified treatment of the outlet boundary conditions since possible physiological mass flow redistribution between the left and right coronary arteries during thrombus growth was not considered. Consequently, the results should be interpreted as a local hemodynamic assessment of thrombus progression near the left coronary ostium.

Since a detailed stent geometry was not explicitly modeled, local hemodynamic effects induced by the stent micro-structure were not captured in the simulations.

Real thrombus morphology may present irregular shapes, eccentric growth patterns, and surface roughness, which are not captured in the present model. Such geometrical variations could locally modify the flow field, wall shear stress distribution, and recirculation regions. Consequently, the current approach represents a simplified scenario and should be interpreted as an idealized approximation. Future studies should investigate more realistic thrombus growth patterns and morphological variations to better represent clinical conditions and assess their influence on the hemodynamic results.

An additional limitation is the decision to neglect the fluid–structure interaction (FSI) effects since the arterial walls and thrombus were assumed rigid. Consequently, possible hemodynamic alterations associated with vessel compliance and thrombus deformability were not considered in the present analysis.

## 6. Conclusions

This study numerically investigated the impact of thrombus formation and growth at the entrance of the left coronary artery, resulting from a misplaced stent. Blood flow within the aortic root and coronary arteries was simulated for several thrombus sizes to evaluate how thrombus growth affects pressure distribution. The results showed that even in the most severe case analyzed—where the thrombus reached 3.1 times its original size—the obstruction of the coronary cross-sectional area was not sufficient to cause a significant pressure drop or wall shear stress increases. This outcome is primarily attributed to the thrombus’s location at the tip of the stent, where its elongation did not substantially reduce the coronary lumen. While the specific configurations studied did not lead to critical obstruction, it is important to note that different thrombus geometries or locations could potentially impede blood flow more severely, resulting in a significant pressure drop in the left coronary arteries.

## Figures and Tables

**Table 1:** Boundary condition type adopted for each case.

Case	Scenario	Input (Aortic Artery)	Output (Right Coronary)	Output (Large Left Coronary)	Output (Small Left Coronary)
1	Without ST	Diastolic pressure [30]	Diastolic Mass flow rate [29]	Diastolic Mass flow rate [29]	Diastolic Mass flow rate [29]
2	Original ST size	Mass flow rate (from Case 1)	Pressure (from Case 1)	Pressure (from Case 1)	Pressure (from Case 1)
3	Elongated ST	Mass flow rate (from Case 1)	Mass flow rate (from Case 2)	Pressure (from Case 1)	Pressure (from Case 1)

**Table 2:** Grid test. Grid Convergence Index.

***Grid Number***	170,000	260,000	430,000
***Grid size*** **(mm)**	0.63	0.54	0.46
***Mesh ratio*** −r	−	1.15	1.18
***Pressure drop*** **(Pa)**$—\Delta P_{lc}$	214	190	191
***Relative Error*** $\varepsilon_{\Delta P_{lc}}$	−	0.126316	0.005236
$GCI_{\Delta P_{lc}}\;(\%)$	−	48.2	1.6
***Inlet pressure at left coronary*** **(Pa)** $—P_{in,lc}$	10,661	10,639	10,641
***Relative Error*** $\varepsilon_{P_{in,lc}}$	−	0.002067	0.000188
$GCI_{P_{in,lc}}\;(\%)$	−	0.79	0.06

## Data Availability

The original contributions presented in this study are included in the article. Further inquiries can be directed to the corresponding author.

## Abbreviations

The following abbreviations are used in this manuscript:

ART	Aortic Root Thrombosis
CFD	Computational Fluid Dynamics
CT	Computational Tomography
LVAD	Left Ventricular Assist Device
ST	Stent Thrombosis
TAVI	Transcatheter Aortic Valve Implantation
WSS	Wall Shear Stress
